# Contrasting effects of rising temperatures on trophic interactions in marine ecosystems

**DOI:** 10.1038/s41598-019-51607-w

**Published:** 2019-10-23

**Authors:** Joël M. Durant, Juan-Carlos Molinero, Geir Ottersen, Gabriel Reygondeau, Leif Christian Stige, Øystein Langangen

**Affiliations:** 1 Centre for Ecological and Evolutionary Synthesis (CEES), Department of Biosciences, University of Oslo, PO Box 1066, Blindern, N-0316 Oslo Norway; 2 Institut de Recherche pour le Développement (IRD), UMR248 MARBEC, IRD/CNRS/IFREMER/UM, Sète Cedex, France; 3 Institute of Marine Research, P.O. Box 1870, Nordnes, N-5817 Bergen Norway; 4 Nippon Foundation-Nereus Program, Institute for the Oceans and Fisheries, University of British Columbia, Aquatic Ecosystems Research Lab, 2202 Main Mall, Vancouver, BC V6T 1Z4 Canada

**Keywords:** Ecological modelling, Phenology

## Abstract

In high-latitude marine environments, primary producers and their consumers show seasonal peaks of abundance in response to annual light cycle, water column stability and nutrient availability. Predatory species have adapted to this pattern by synchronising life-history events such as reproduction with prey availability. However, changing temperatures may pose unprecedented challenges by decoupling the predator-prey interactions. Here we build a predator-prey model accounting for the full life-cycle of fish and zooplankton including their phenology. The model assumes that fish production is bottom-up controlled by zooplankton prey abundance and match or mismatch between predator and prey phenology, and is parameterised based on empirical findings of how climate influences phenology and prey abundance. With this model, we project possible climate-warming effects on match-mismatch dynamics in Arcto-boreal and temperate biomes. We find a strong dependence on synchrony with zooplankton prey in the Arcto-boreal fish population, pointing towards a possible pronounced population decline with warming because of frequent desynchronization with its zooplankton prey. In contrast, the temperate fish population appears better able to track changes in prey timing and hence avoid strong population decline. These results underline that climate change may enhance the risks of predator-prey seasonal asynchrony and fish population declines at higher latitudes.

## Introduction

Climate variability shapes physiological traits, spatial distribution and interactions among species, defining the phenology (i.e., seasonal timing of cyclical biological events such as reproduction), the trophodynamics and ultimately the pace of matter and energy transfer in food webs^1^. Species have evolved complex behavioural and life history strategies to maximize fitness, e.g. exploiting the periods of the year best matching optimal niche requirements for growth, survival and reproduction^2,3^. Thus, at long time-scales evolution shapes the distribution and the phenology of predators and prey^4,5^. Species below the top predator level engage in a co-evolutionary game^6^ consisting in seasonally matching their prey while “mismatching” their predators^7^.

The match-mismatch hypothesis (MMH) has long been considered one of the seminal principles towards explaining the variability of recruitment in fish populations^5,8–10^, proposing that inter-annual changes in a predator’s growth and survival depend on the degree of match between its food requirement and prey availability. The phenology of predators and prey, and thereby the degree of match or mismatch, are constrained by temperature-dependent limitations, which are particularly strong for ectothermic organisms, such as fish and their prey. Hence, any change in climate may trigger unexpected responses unbalancing established patterns in trophic interactions, ultimately affecting the recruitment of higher trophic levels^5^.

Global climate change affects both the abiotic and biotic compartments of marine ecosystems, but with marked variations across taxa, functional groups and ocean regions^11,12^. In particular, sea temperature changes may have significant influence on populations through mechanisms ranging from basic physiological processes^13^ to changes in distribution of both zooplankton and fish species^12,14^ and whole biological communities^15,16^.

In this paper the focus is on how warming affects marine species phenology^17^ and thereby trophic interactions^2,18^. Future changes in predator-prey interactions are generally elusive, as they depend on a complex interplay between physiological based constraints and feedbacks within and between species, leading to uncertain projections of biological effects of climate change. A potential phenological mismatch between lower and higher trophic level species is however of utmost importance in structuring marine food webs, as it could not only negatively impact the productivity, but also enhance vulnerability of harvested fish^19^.

The effect of climate change on match-mismatch dynamics is likely to depend on latitude, as the degree to which biological processes such as photosynthesis and visual feeding are seasonally constrained and typically vary with latitude. Here we aim to enhance the understanding of the similarities and differences between temperate biomes (TB) and Arcto-boreal biomes (AB) in how the trophic coupling between pelagic marine fish populations and their zooplankton prey may respond to climate change. This understanding is crucial as it might shed light on the potential for the productivity of these regions.

## Results and Discussion

**Figure 1:**
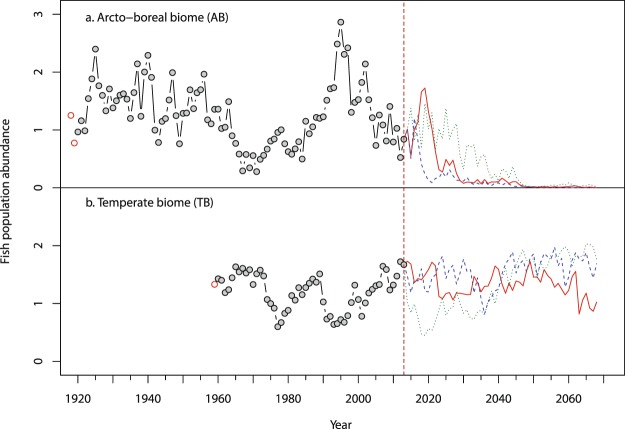
Temperature and match-mismatch effects on the fluctuations of the fish populations. Simulation and projection of the change of the theoretical fish population following the change of strength of the match-mismatch relationship as driven by environmental conditions. Simulation (before 2013, dots, with red circles showing initial values) and projection (3 different runs; run 1 = plain red, run 2 = dotted green and run 3 = dashed blue line, all starting in 2013 and running for 50 years) of the population change for (**a**) an Arcto-boreal fish in the Barents Sea between 1921 and 2063 and for (**b**) a temperate fish in the Bay of Biscay between 1960 and 2063. The projection was done using the climatic changes projected by the CMIP5 simulations of the Max Planck Institute for Meteorology (MPI-M) based on the MPI-ESM-LR model (RCP 4.5 experiment, 3 runs). The length of the simulation depends on the length of the data available (Supplementary Table S1). Note that the three runs deviate due to the natural variability in the physical environmental projections.

**Figure 2:**
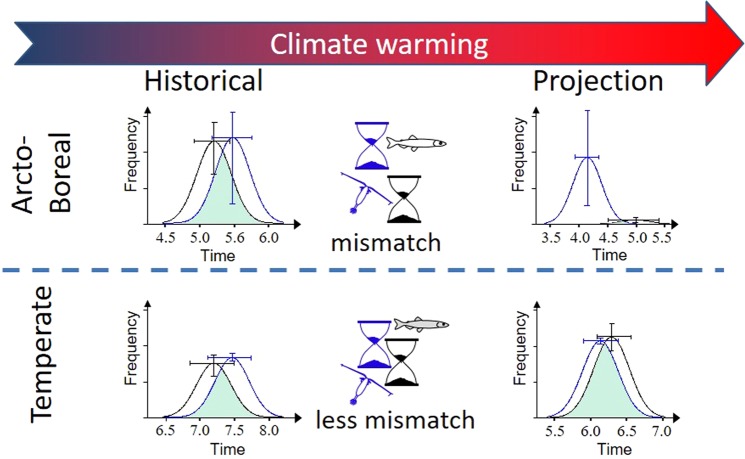
Effect of climate change on match-mismatch and population change in two different biomes. The curves show mean hypothetical seasonal trends in food requirements of a predator population’s offspring (black) and the abundance of their prey (in blue) for the two biomes for historical (1980–2000) and future (2040–2060) periods. The bars show the interannual variability (the 0.2 and 0.8 quantiles). The x-axis is in months and the y-axis in arbitrary unit. The total food requirement (the area under the black curve) is assumed to scale with the abundance of adult predators (Supplementary methods). The overlap between the curves (green shaded area) gives an indication of the reproductive success of the predator, with larger overlap indicating stronger recruitment to the predator population^9^. A decrease of temporal asynchrony and/or an increase of prey relative to predator abundance increase overlap.

The 50-year projection (Figs 1 and 2) shows a contrasting response to climate change for the pelagic fish species living in the TB contra the AB. The AB fish population is projected to decrease in abundance while the TB population is projected to remain stable. Comparing two 20-year periods, 1980–2000 vs. 2040–2060 under the RCP 4.5 emission scenario, the AB shows a future decrease of temporal overlap between fish and prey because of earlier occurrence of prey in spring while the fish requirement timing remains relatively constant during the year (Fig. 2). By contrast, for the TB both the predator and prey have advanced timing, leading to maintenance of the temporal overlap (Fig. 2).

One of the highest uncertainties in projecting effects of climate change using ecosystem modelling resides in the quantification of the nature and strength of species interactions^20^. Climate change may have unforeseen consequences because responses to environmental changes differ among marine species and trophic levels, and also across ocean regions^11,12^. For instance climate change may lead to a decoupling between the lower (primary producers and consumers) and higher trophic levels (secondary and apex predators)^21^. Moreover, climate related shifts interact with other stressors^22,23^, i.e. exploitation, thereby amplifying the impacts on ecosystem functioning. For instance, fishing induced age–size truncation increases the population sensitivity to phenological mismatch, ultimately enhancing abundance variability in harvested fish^24^. It is therefore crucial to gain insights into which regions and trophic levels such decoupling might be the most pronounced, in order to develop effective management policies to face the impact of future ecosystem changes on fisheries^25^.

Environmental conditions, including seasonality, are assumed to drive macro-ecological patterns of marine diversity, abundance and ecosystem functioning by shaping the biological characteristics of species through evolution^26^. Indeed, species niche breadth appears to vary with latitude, displaying large plasticity in the temperate biomes associated with a cosmopolitan capacity, while a narrow environmental tolerance in tropical and polar regions reflects specialisation^27^. In a context of climate warming, ectothermic species that represent the majority of marine diversity, with a narrow environmental niche, closely track the velocity of climate change, as revealed by changes in phenology, local extinction or poleward migration^12,15,17,28^. Sensitivity analysis of our model shows that during the historical period an increase of temperature was associated with an increase of abundance for the high-latitude AB species, while the temperature effect was weaker for the mid-latitude TB species (Supplementary Table S2). Yet, our results also suggest that the AB species is strongly negatively affected by changed phenology if temperature increases beyond the historical range (Supplementary Fig. S1). This was because the projected warming led to more frequent mismatch situations and recruitment failure of the AB fish (Supplementary Fig. S2), consistent with high thermal specialisation of Arctic species (Fig. 1 and^27^). Already during the historical period, the distribution of the match-mismatch situations is different between AB and TB (Supplementary Fig. S2). AB more often shows situations with full match or full mismatch (extremes) than TB. This pattern becomes even more pronounced during the projection period with over 50% of the years displaying a full mismatch and hence a very low production of young indivivuals. This scenario was envisioned by Cury *et al*.^21^ as a response to a change in the amplitude of year-to-year variations in prey timing in regions where interannual variability in temperature is expected to increase. The strong population decrease described by our model can be attributed to a too frequent lack of recruitment. While temperature increase leads to an earlier phenology for both plankton and fish in both systems, the impact is of different intensity with stronger effect on AB than TB fish (Supplementary Table S2). In AB, the reason for the modelled mismatch was an early peak timing of the plankton (about 1.2 months, Fig. 2) that was not matched by the fish (Fig. 2). Of the components determining the peak timing of larval feeding, the spawning date for the AB fish increased in variability and the median spawning date shifted to about 0.4 months later in the season in the projected future, while the hatching time decreased by approximately 0.4 months, the yolk resorption duration being similar for both periods. The projected timing of larval feeding for the AB fish thereby only changed slightly in response to the increasing temperatures. The TB species, in contrast, appeared to sustain a projected temperature increase beyond the historical range by adjusting the timing of their reproduction to temperature and hence prey availability, suggesting that temperature effects on prey abundance and timing are unlikely to limit the realised thermal niche. In other words, our results shed light on how match-mismatch dynamics may restrain the realised thermal niches of AB fish more than of TB fish, which is consistent with the general pattern of narrower thermal niches for marine fish in polar regions in contrast to temperate regions (see Fig. S4 in^27^).

Our results support the expected outcome that high-latitude pelagic ecosystems (i.e., over the polar circle) may be particularly vulnerable to phenological changes caused by climate warming. Here, we show that recruitment success of higher trophic levels can be highly dependent on synchronisation with seasonally pulsed primary production, while the response to regional warming varies among functional groups. This fits with the established understanding claiming that match-mismatch dynamics become progressively more important toward higher latitudes, as the period available for phytoplankton and zooplankton production decreases with latitude due to increasing restrictions in light and nutrients. Contrarily, the higher stability in light level and biological productivity in the lower latitudes moderates the importance of match-mismatch^29,30^. Hence, as shown by our results, climate change is expected to affect the “rules” of the co-evolutionary game between predator and prey by desynchronising, at an unknown rate, the higher trophic levels from the pulsed planktonic production.

Species-specific phenological shifts caused by climate warming may have wide-ranging consequences. Based on a multispecies match-mismatch perspective, Nakazawa and Doi^31^ suggested that phenological synchrony among interacting species also affects key dynamical features of whole communities. In particular, our results warn of ecosystem-wide changes in the AB, as the modelled trophic level, i.e. pelagic small fish, is at a pivotal position linking primary and secondary producers with the higher trophic levels. Note that Arcto-boreal biomes are composed of relatively few numbers of species thus giving each species a pivotal role, since a loss in their abundance is less likely to be compensated (in terms of function) by other species. In contrast, warming is not found to lead to desynchronization in the TB food web, as the phenology of the fish and the phenology of the plankton^32^ respond similarly to temperature (Fig. 2). Here, the fish respond to higher temperature both by spawning earlier, i.e., when temperature reaches 17 °C^33^, and by faster egg and larval development. In the AB, temperature warming leads to mismatch because the phenology of the fish is not keeping track with the changes in plankton phenology (Fig. 2). Indeed in the AB, the fish respond to higher temperature by faster egg and larval development, but not by earlier timing of spawning, which takes place in our model when winter temperature starts to increase. We therefore suggest that such a difference, possibly genetically determined^34^, reflects a general pattern of higher seasonal constraints on spawning phenology in high-latitude environments. Our results support that high-latitude species may be particularly sensitive to phenological shifts under climate change^2,11,18^. Our modelling approach, i.e., a fairly simple and transparent model based on established empirical relationships, gives the theoretical basis for a mechanism that may reduce productivity at high latitudes that does not yet occur at temperate latitudes.

We have of practical necessity as well as for transparency made some simplifying assumptions for the analysis. We focused on one mechanism, match-mismatch, and not on all aspects linking sea temperature to fish population dynamics. Hence, we do not claim that Arcto-boreal fish populations generally will do poorer than temperate populations under climate change, but point to the expected influence of trophic mismatch from future warming.

The chosen modelling approach favours generality over precision, causing the need for some caution when interpreting the results. For instance, we do not explicitly model effects of light, which may directly or indirectly constrain both zooplankton and fish phenology. The projected advancement of zooplankton timing in the AB (Fig. 2) is based on historical associations with temperature^35^. These associations are supported by more recent analyses^36^ and longer time series^37^, and are consistent with earlier phytoplankton blooms and earlier reproduction and faster developmental rates of zooplankton in warm waters^38^. In a warmer climate, the AB zooplankton abundance is projected to start increasing from mid-March (Fig. 2), although it cannot be ruled out that low light rather than direct or indirect effects of temperature may limit phytoplankton growth, zooplankton feeding, reproduction and survival in the AB at this time of year. In contrast, the TB zooplankton are in a warmer climate projected to start increasing in abundance from late May, when light limitation seems unlikely.

Furthermore, the empirical knowledge about seasonal constraints on fish spawning remains sparse. Our empirical-based approximation of mean spawning date for the AB is defined as the time when the temperature starts to increase in spring (Supplementary Fig. S3). This is a simplification since the spawning time is, e.g., likely influenced by the temperature experienced by the spawners during the whole length of the reproductive cycle^39^. However, the effect of climate warming on spawning time is not straightforward as the fish may migrate both horizontally or vertically to stay within a preferred temperature range. This is especially true for migratory pelagic fish, such as the AB capelin, which feeds near the polar front^40^. During warm years the mature capelin feed farther north, not necessarily in warmer temperatures, and possibly only experience increased temperatures when migrating towards the coast in late winter and early spring. Accurate prediction on how climate change may affect spawning behaviour of this and other key marine fishes requires continued building of relevant long-term data and model studies to help elucidate the underlying processes.

The model used is parsimonious and does not include a stochastic term. However, adding such terms for juvenile and adult survival did not change the overall outcome of the projection (Supplementary Fig. S4). Other mechanisms that can create yearly variation of juvenile mortality were not explicitly considered, although we assumed such mechanisms to be implicit in the survival term in the simple stock recruitment relationship we used (Table 1). For instance, we did not account for the variations in predation rates, which at high latitudes may increase with temperature^16^. While we did not model the potential density-dependence effect on the egg production or adult survival, we took density-dependence into account for the larval survival with the overlap calculation, done in a similar way as in the Dynamic Bioclimate Envelope Model^41^. Likewise, as the match-mismatch hypothesis was developed for bottom-up controlled systems, our model did not consider possible negative effects of fish abundance on plankton. Our model assumes that food requirement and prey availability over time follow a normal distribution^9^, which is not necessarily appropriate; increased frequency of autumn phytoplankton blooms^42^ may, for example, lead to more skewed zooplankton distributions by inducing the zooplankton to remain active for a longer time in the season. This hypothesis however, did not affect our main conclusions (decrease to collapse for the AB fish and maintenance of high abundance for the TB fish) as shown when using a log-normal distribution model (Supplementary Fig. S5). Lastly, to ensure that our model tested the effect of match-mismatch relationship correctly we ran a simulation with full time synchrony (Supplementary Fig. S6) and found in such case a higher fish abundance, while most of the variation disappeared for the TB. This confirmed that the asynchrony between plankton and fish is limiting the fish recruitment, recruitment being the only variable affecting the population abundance, mortality being maintained constant in our model. In spite of the above caveats, our approach provides insight in the potential mechanisms behind the observed effect of climate on abundance through change in phenology^2,11,18^.

**Table 1:** Summary of the model used for the simulations of MMH interaction.

Time step	Arcto-boreal fish (2 years to be recruited)	Temperate fish (1 year to be recruited)
t	*overlap*_t_ = f(m_fish,t_, m_plk,t_, N_fish,t_, N_plk,t_, s_fish,t_, s_plk,t_)/N_fish,t_	*overlap*_t_ = f(m_fish,t_, m_plk,t_, N_fish,t_, N_plk,t_, s_fish,t_, s_plk,t_)/N_fish,t_
t + 1	n_t+1_ = *a*·N_fish,t_ · *overlap*_t_	n_t+1_ = *a*·N_fish,t_ · *overlap*_t_ N _fish,t+1_ = n_t+1_ + N_fish,t_ · 0.5
t + 2	N_fish,t+2_ = (n_t+1_ + N_fish,t+1_) · 0.5	

Subscript t refers to year. f = function calculating the overlap between 2 normally distributed curves defined by m (time of the peak of fish offspring food requirement or zooplankton abundance), N (abundance of adult fish or zooplankton), s (standard deviation that is assumed constant and equal to 0.25) and *a* (factor linking N to n estimated to be equal to 2.27 for AB fish and 0.8 for TB fish); see Supplementary methods and^9^. Note that here we assume that the food requirements of a predator population’s offspring scales with the abundance of adult predators N_fish_. The result is divided by N_fish,t_ to scale the overlap between 0–1. ‘n’ is immature fish abundance. The equations and the data source used to estimate m and N are found in the Tables 2 and S1.

0.5 is the survival from step t to step t + 1 and assumed to be the same for immatures and adults (but see Supplementary Fig. S4).

While our results suggest a potential population collapse in the AB population, it could persist if capable of rapid adaptation to the changes in prey phenology. For example, shifting prey preferences could allow persistence, if suitable alternative prey are, or become, available. A reduction of only 40% of the dependence of juvenile survival to its prey phenology/abundance (providing no other density dependence mechanism) would be enough to prevent the breakdown (Supplementary Fig. S7). In addition, the AB fish could potentially shift their dominant spawning period from spring to summer to match the production associated with the autumn phytoplankton bloom occurring more frequently with climate warming^42^. Lastly, we acknowledge that ectotherms often mature earlier at higher temperatures^43^. However, we note that reproduction at a younger age does not seem to secure population persistence, as shown by our simulations using a 1-year instead of a 2-year life cycle (Supplementary Fig. S8). Analogously, while our results suggest that the TB fish may be robust to temperature-driven mismatch with prey in a warmer climate; this finding is no guarantee for population persistence. Rather our results suggest that other mechanisms may be more important for future population trajectories of TB fish, such as direct physiological temperature effects and changes in predation regime.

Finally, our results are consistent with the expected larger sensitivity of the Arcto-boreal region to climate change induced by phenological change^11^. There is no contradiction between our findings, i.e. the potential future negative effect of rising temperatures through mismatch between prey and predators, and other potential positive effects of climate-change on Arcto-boreal species^15,16^. Indeed, our modelled AB fish responded positively to increased temperatures within the historical temperature range (Supplementary Table S2), although future warming may have adverse population effects. Our results provide valuable insights into differential phenological responses in two climatic biomes that can foster ecosystem-wide shifts with implications for biodiversity, trophic pathways and ultimately ecosystem services.

## Methods

To investigate the effect of climate driven phenological changes on trophic interactions, we built a predator-prey model that integrated our current knowledge about how climate influences the phenology of planktivorous fish and their zooplankton prey at different latitudes. We assumed that the fish recruitment depended on the degree of overlap phenological match-mismatch^5,8^ between the food requirements of the fish larvae and the abundance of its prey, i.e., on the phenology of both predator and prey, as well as the abundances of the fish and the prey. The model was parameterised for a mid-latitude temperate biome and a high-latitude Arcto-boreal biome. Using this model and climate projections provided by IPCC (Intergovernmental Panel on Climate Change, http://www.ipcc-data.org) we projected effects of match-mismatch on the populations in the two biomes over the next 50-year period.

### Overlap between predator and prey as proxy of recruitment success

**Figure 3:**
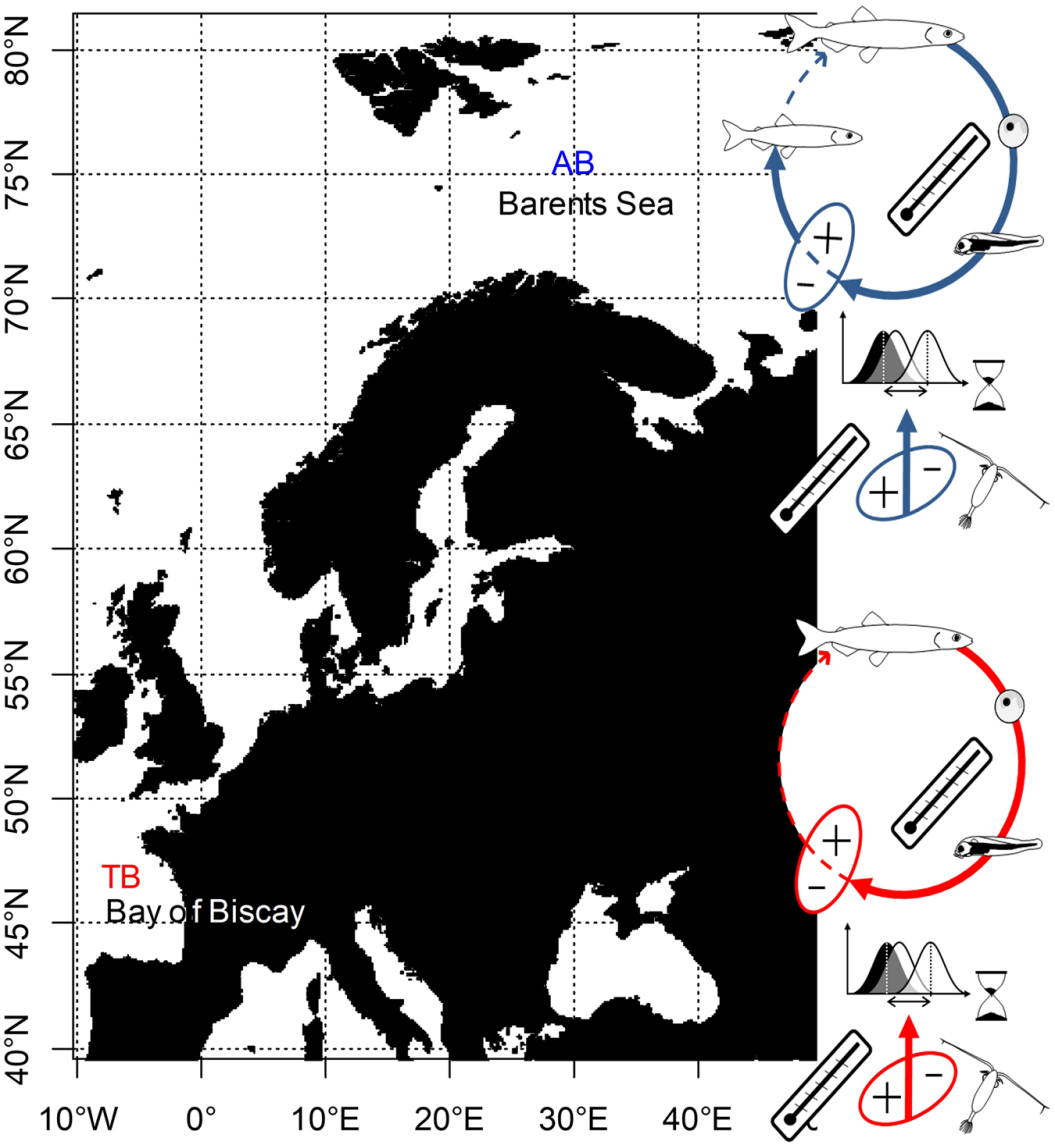
Study area and schematic presentation of the life cycles used. To simulate the change in fish species abundance with the change in degree of synchrony between a prey (plankton) and a predator (fish) we followed the match-mismatch hypothesis^9^. We assumed that the Arcto-boreal species (first row, blue) has a longer life cycle than the temperate one (second row, red). AB is for Arcto-boreal biome and TB for Temperate biome.

We modelled the changes in abundance of small pelagic fish with recruitment bottom-up controlled by their plankton prey in a match-mismatch relationship. Two populations were modelled; one population represented an Arcto-boreal biome (AB) and the other a temperate biome (TB), Fig. 3. We chose to model small pelagic fish because of their relatively low position in the trophic chain and their relatively short life cycle, making them more sensitive to recruitment variability^44^ and to effects of climate change on recruitment. Here we used the approach of Durant *et al*. 2005, considering that the overlap between the curves of frequency of the predator and its prey is a proxy for the predator success, i.e. young age survival to recruitment (Fig. 3 and Supplementary Methods). The rational of the assumption is that a high overlap indicates a high possibility of feeding for the predator (Fig. 3). This overlap changes as a function of the synchrony of the two distributions, which depends on timing of spawning and development rates, as well as the relative abundance of prey to predator^9^. Specifically, a high overlap, i.e., *overlap* ≈ 1, corresponds to a match situation^9^ with high recruitment rate while a low overlap, i.e., *overlap* ≈ 0, a mismatch with poor recruitment.

### The match-mismatch model

For our model we needed information on the phenology and abundance of a small pelagic fish and its prey for the two biomes. The relationships between climate variables and abundance and phenology were extracted from empirically-based work on a key pelagic fish and its main prey for each biome^35,45–48^. For both biomes the environmental variables and their relationships to model parameters are given in Supplementary Table S1 and Table 2 respectively (see also Supplementary Fig. S9).

**Table 2:** Summary of the equations used in the model.

System	Equations used in the match-mismatch model	Ref
Arcto-boreal biome	m_plk,t_ = (176.6 – 17.55 · *TEMP*_*SPR*,t_)/365 · 12 + 1.45	^a,†^
	N_plk,t_ = exp(0.02 + 0.13 · NAO_t_ + 0.58 · *TEMP*_*ΔSUM*,t_) − 1	^b^
	m_fish,t_ = Spawning + 1/(0.0092 + 0.0051 · *TEMP*_*spawn* t_)/30 + (21.25 · exp (−0.083 · *TEMP*_*hatch* t_)/30	^c^
Temperate biome	m_plk,t_ = (602.74 + 2.34 · NAO_t_ − 24.87 · *TEMP*_*BB* t_)/365 · 12	^†^
	N_plk,t_ = 0.19 · *TEMP*_*BB* t_ − 0.01 · NAO_t_ − 1.84	^‡^
	m_fish,t_ = Spawning + (661.1 · *TEMP*_*spawn* t_^−2.08^)/30 + 21.25 · exp (−0.083 · *TEMP*_*hatch* t_)/30 + 0.56	^!!^

Subscript t refers to year.

*TEMP*_*spawn*_ = mean temperature during the 2 months following Spawning date. *TEMP*_*hatch*_ = mean temperature during the 2 months following Hatching date.

^a^Ellertsen *et al*. 1989^35^, Assuming 1990 was a good year and represented a full match situation we adjusted m_plk_ by adding 1.13 in order to obtain m_fish,1990_ − m_plk,1990_ = 0.

^b^Stige *et al*. 2014^45^.

^c^Duration from spawning to hatching = 1/(0.0092 + 0.0051 · *TEMP*_*spawn*_) in days^48^, and duration from hatching to full yolk resorption = 21.25 · exp (−0.083 · *TEMP*_*hatch*_) in days^46^. Both are divided by 30 to get the value in months.

^†^Divided by 365 days and multiplied by 12 to get m in the scale of months.

^‡^Model for *Centropages typicus* abundance in the Bay of Biscay between 1972–2012 (see Supplementary methods).

^!!^Duration from spawning to hatching = 661.1 · *TEMP*_*spawn*_^−2.08^ in days^46^, and duration from hatching to full yolk resorption = 21.25 · exp (−0.083 · *TEMP*_*hatch*_) in days^46^. Both are divided by 30 to get the value in months. Assuming 2011 was a good year and represented a full match situation we adjusted m_fish_ by adding 0.56 in order to obtain m_fish,2011_ − m_plk,2011_ = 0.

The modelled zooplankton population in the AB represented a copepod-like species using published relationships for *Calanus finmarchicus* in the Barents Sea. The AB fish population was modelled based on published relationships for the capelin *Mallotus villosus* and assumptions on spawning time. We considered, by looking at reference^47^ (see Supplementary Fig. S3), that the mean spawning date is defined each year as the time (in month) when temperature starts to increase using a 2^nd^ order polynomial model on the monthly TEMP values (sea temperature: see Table 2). Spawning before mid-February (in month 2.5) was not considered possible due to light conditions in the Barents Sea and was replaced by the average of the 20% lower values (3 occurrences between 1921 and 2060).We considered that the fish take two years to mature from spawning (Fig. 3 and Table 1). While the capelin is essentially semelparous, we did not limit the number of reproductive events of our generic AB fish. However, the adult survival used was an average of the survival of the reproducing and not reproducing adults (>2 years of age), and the mean life expectancy of our generic species (on average 4 years) was similar to that of capelin.

The zooplankton population in the TB was modelled using biological relationships for *Centropages typicus* in the Bay of Biscay. The temperate fish population was modelled using published relationships for the European anchovy *Engraulis encrasicolus* and assumptions on spawning time. We considered that the mean spawning date is defined each year as the time (in month) when TEMP becomes >17 °C (see Fig. 2 in^33^ showing that 50% of the eggs are laid when TEMP reach 17 °C) using a 2^nd^ order polynomial model on the monthly TEMP values. We considered that the fish take one year to mature (Fig. 3 and Table 1), while we did not put any limitation on the number of reproduction attempts. The maximum age reached was defined by the survival. Considering that the larvae mortality is mainly due to mismatch with their prey (already included in the model) we did not add to the model a mortality term for the immatures (n in Table 1).

### Model and simulation of the fish population change

The equations used to calculate the change in fish abundance are given in Tables 1 and 2. We considered that the juveniles surviving to year t + 1 (n_t+1_) produced by adults in year t (N_fish,t_) are match-mismatch dependent on a plankton population (N_plk,t_). We assume that there is no density-dependence at the egg production, but food-driven density-dependence in survival of the offspring. In other words, we assumed that offspring production is directly proportional to the number of adults (*a*·N_fish,t_), while the variation of survival of the young due to density-dependent effects (large stock and/or low prey abundance) is taken into account by multiplying by the *overlap* (Table 1). The value for *a* was selected in order for the *overlap* variance to be similar to the observed variance in recruits per spawner (Supplementary methods).

Since the initial values were unknown, we used values taken from the stable population for this purpose. We estimated the initial values with a 20-year “pre-simulation”. For this we started respectively for AB and TB in 1901 and 1940 with as starting parameters N_fish_ = N_plk_ = 1 (arbitrary units), m_plk_ = m_fish_ = 1 (month scale) and s_fish_ = s_plk_ = 0.25 month. We let the values of N and m change through time following the simulation model as described in Table 1. The environmental variables used for this 20-years pre-simulation were randomly picked up from the observed pair of winter North Atlantic Oscillation (NAO) and temperature values between 1921–2013 and between 1960–2013. We then performed a 1000 step bootstrap to estimate the average N_fish_ and N_plk_ for the 4 years before 1921 in the AB and 2 years before 1960 in the TB system (the AB fish having a longer life cycle than the TB fish, Fig. 3).

We then ran the simulation model using these initial values for N_fish_ and N_plk_ and the observed environmental variables for the historical periods (i.e. for AB 1921–2013, for TB 1960–2013). The results of the simulation are shown in Fig. 1.

Analyses of sensitivity to the change of environmental variable values were conducted for the historical periods by increasing by ½ a standard deviation the variable to test (temperature, NAO, N_plk_) and looking at the effect of this increase on the corresponding simulated N_fish_ (Supplementary Table S2).

### Projection of climate change effect on the fish populations with match-mismatch driven recruitment (2013–2063)

To project population changes over the next 50 years for the Barents Sea and the Bay of Biscay we applied output from the MPI-ESM-LR of the Max-Planck-Institute for Meteorology (MPI-M) in Hamburg (http://cera-www.dkrz.de/WDCC/ui/)^49^. This is one of the Earth system models (ESMs) used within the IPCC’s Fifth Assessment (AR5) Coupled Model Inter-comparison Project 5 [CMIP5].

We used the medium-emissions trajectory scenario RCP4.5 (Representative Concentration Pathway 4.5, radiative forcing of 4.5 W m^−2^ at year 2100 relative to pre-industrial conditions). The data (3 runs for the same scenario) represent monthly averaged values of selected variables.

To estimate the change in different temperature variables needed for the projections we used satellite-measured “skin” ocean surface temperature (for AB between 70–73°N and 30–35°E, for TB between Cap Ortegal (43°46′N 7°52′W) and Penmarch Point (47°48′N 4°22′W)) and adjusted the obtained results to the observed measurements (Supplementary Fig. S1). To estimate the changes in NAO we used the normalised difference in sea level pressure between two areas within 90°W–40°E, respectively 20–40°N for “the Azores” and 50–80°N for “Iceland”, for the months of December through March^50^.

## Supplementary information

Supplementary materials

## Data Availability

All previously published data are available online (references given), other data given in the Supplementary material.
