# Supplementary material for: Contrasting effects of rising temperatures on trophic interactions in marine ecosystems
Source: Sci Rep. 2019 Oct 23;9:15213. doi: 10.1038/s41598-019-51607-w (PMC6811528; doi:10.1038/s41598-019-51607-w)
Supplement: Supplementary file 1 — Supplementary materials [file 41598_2019_51607_MOESM1_ESM.pdf]

Supplementary information for

Contrasting effects of rising temperatures on trophic interactions in marine ecosystems

Joël M. Durant, Juan-Carlos Molinero, Geir Ottersen, Gabriel Reygondeau, Leif Christian Stige and Øystein Langangen

Running head: "Match-mismatch and climate warming"

Supplementary methods

I. Predator-prey overlap

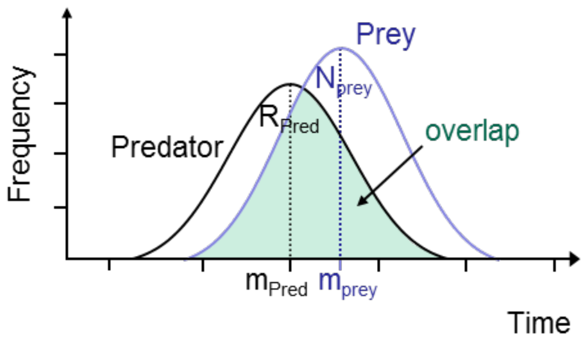

Match-mismatch model following Durant et al. 2005. The figure shows hypothetical seasonal curves for the food requirements of a predator population's offspring (R_{Pred} in black) and the abundance of their prey (N_{prey} in blue). The total food requirement (the area under the R_{Pred} curve) is assumed to scale with the abundance of adult predators, N_{Pred}. The overlap between the curves (green shaded area) gives an indication of the reproductive success of the predator, with larger overlap indicating stronger recruitment to the predator. N and R = surface delimited by the curve or abundance, m = time of the peak of abundance, t = time and s = standard deviation. Note that standard deviations of the curves (s_{Pred} and s_{prey}) are not indicated

Following the classical graphic match-mismatch representation (Cushing 1990; Durant et al. 2005; Durant et al. 2013; Durant et al. 2007), we assumed that the food requirements of a predator population (fish) and the abundance of available prey (plankton, plk) over the season (x-axis) each follow unimodal bell-shaped distributions (y-axis; Gaussian distributions; Fig. S1). The success of the upper trophic level (i.e., young age survival to recruitment) is thus assumed to be proportional to the degree of overlap (later *overlap*) between the two curves (defined as the fraction of the area under the predator curve that overlaps with the area under the prey curve) in a classical bottom-up process. This overlap changes as a function of the synchrony of the two distributions as well as the relative abundance of prey compared to predators (Durant et al. 2005).

The normally distributed seasonal curves (frequency see fig. A1) can be written as:

$$\text{frequency} = \mathrm{N}/(\sqrt{2\pi} \cdot s) \cdot \exp[-\frac{(tm-m)^2}{2s^2}]$$

with N = surface delimited by the curve, in our case representing either the total food requirements of the juvenile fish or the abundance of plankton, m= time of the peak (scale: decimal months, 1,...,12), tm = time (months) and s = standard deviation (months). The total food requirements of a year-class of fish offspring is assumed to scale with adult fish abundance, N_{fish}; hence N_{fish}, is used as numerator N for the fish equation. The recruitment of juvenile fish (n_{fish}) scales with the product of the total food requirements (and hence N_{fish}) and *overlap* (see below). Change in N_{fish} depends on recruitment and adult survival (table 1). N for the plankton (N_{plk}) and m (m_{plk} and m_{fish}) are modelled to depend on climate variables based on relationships found in the literature (Table 2) and s (s_{plk} and s_{fish}) is considered constant over the years and equal to 0.25 (1 month duration). We calculated by numerical integration the *overlap* every year (time step t, see R codes). To ensure that each $overlap_{\ t}$ is ranging from 0 to 1 it was divided by $N_{fish,t}$. A high *overlap*, i.e., ≈1, corresponds to a match situation (Cushing 1990; Durant et al. 2005) with high recruitment rate. When the *overlap* is low, i.e., ≈0, there is a mismatch and the recruitment is low.

R code used to compute the overlap under two Gaussian curves

```
overlap <- function(mplk,mfish,Nplk,Nfish,splk,sfish) {
	# With m = time of the peak of abundance, N = abundance and s = standard deviation
	for plk=prey and fish=predator
	# the idea is to approximate the overlap surface by a high number of polygons, to
	calculate the surface of each polygon and sum them.
	sdMAX<-3*max(c(splk,sfish),na.rm=T)   # define the maximum width of the curves
	mMAX<-max(c(mplk,mfish),na.rm=T)    # define the maximum time m
	mMIN<-min(c(mplk,mfish),na.rm=T)     #  define the minimum time m
	tt1<-seq(mMIN-sdMAX,mMAX+sdMAX,((mMAX+sdMAX)-(mMIN-
		sdMAX))/(10000+5000*abs(mplk-mfish)))  # build a sequence of values for
		the whole time period covered by the two curves. The sequence length if of
		ca. 15000 values.
	delta<-((mMAX+sdMAX)-(mMIN-sdMAX))/(10000+5000*abs(mplk-mfish)) # define
the width of each polygon

	f1<-Nplk /(sqrt(2*pi)*splk) *exp(-(tt1- mplk)^2/(2*splk^2))    # Gaussian curve for the
plankton/prey. Will be used to calculate the height of the polygon
	f2<-Nfish/(sqrt(2*pi)*sfish)*exp(-(tt1- mfish)^2/(2*sfish^2))   # Gaussian curve for the
fish/predator. Will be used to calculate the height of the polygon

# add the next code to visualize the curves

	b<-which(f1-f2<0) # define which part of f1 is overlapping with f2
	c<-which(f2-f1<0) # define which part of f2 is overlapping with f1
	overlap<- sum(f1[b]*delta)+ sum(f2[c]*delta) # sum the polygons surfaces
 }
```

example using the following values:

```
mplk=1.6
splk=0.5
Nplk=1
mfish=1
sfish=0.5
Nfish=1
a<-overlap(mplk,mfish,Nplk,Nfish,splk,sfish)
a
```

R code to visualise the Gaussian curves

to include in the previous R code where indicated

```
plot(tt1,f1,type="l", ylim=c(0,max(c(f1,f2),na.rm=T)),col="dark
        green",ylab="frequence",xlab="Time")# plankton/prey in green
        lines(tt1,f2, col="red")                    # fish/Predator in red
        lines(c(mplk,mplk),c(0,max(f1,na.rm=T)),lty=2,col="dark green")
        lines(c(mfish,mfish),c(0,max(f2,na.rm=T)),lty=3,col="red")
```

II. Phenology and abundance modelled for *Centropages typicus* in the Bay of Biscay (Table 2).

Factor	Indices considered	Description and source
Centropages typicus phenology	$m_{plk,\ t}$	Data were obtained from the CPR programme operating in the Bay of Biscay over the period 1970-2012
Centropages typicus abundance	$N_{plk,\ t}$	Data were obtained from the CPR programme operating in the Bay of Biscay over the period 1970-2012
Annual temperature	$TEMP_{BB\ t}$	Mean BB temperature for and Jan $_t$ to Dec $_t$ (see Table S1)
North Atlantic Oscillation	NAO_t	Station based winter (Dec $_{t-1}$ – March $_t$) NAO index (see Table S1)

The in situ ecological information was acquired from the Continuous Plankton Recorder (CPR), which is an upper layer plankton-monitoring programme running in the North Atlantic since 1931 (Reid et al. 2003).

To explore the relationship between the timing of *Centropages typicus* seasonal peak and NAO and temperature, we used the median of the month (e.g. day 15) in which the annual peak of the copepod abundance was observed. Thus, if the annual peak occurred in July, the corresponding day of year was = 196).

Prior the statistical analysis, copepods data were log transformed and standardized to zero mean and unit variance. Phenology changes in *C. typicus* were investigated by general linear models (GLMs) that were run separately to assess the contribution of each predictor variable to copepod phenology and abundance. Partial regression coefficients were considered significant when $p<0.05$.

1. Phenology model of Centropages typicus in the Bay of Biscay over the period 1970-2012

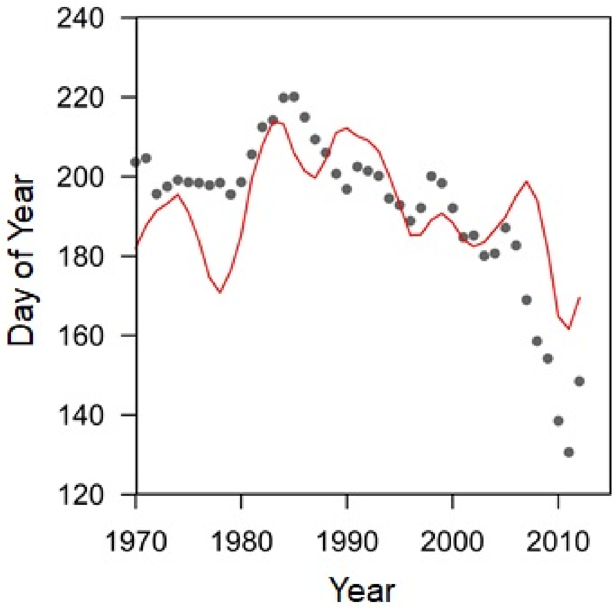

Year-to-year changes in the phenology of *Centropages typicus*. Comparison of the observed data, in dots, and the model output (see following equation and table), red continuous line.

$m_{plk,\,t} = (602.74 + 2.34 \cdot NAO_{t} - 24.87 \cdot TEMP_{BB\,t})$

Coefficients of the model *Centropages* phenology as function of NAO and SST.

	Param.	Std Error	t	p
Intercept	602.74	177.31	3.40	0.00
NAO	2.34	2.72	0.86	0.40
TEMP	-24.87	10.43	-2.38	0.02

Explanatory ability of the model

R	R^2	$F_{2,29}$	p
0.40	0.23	3.01	0.05

2. ***Monthly abundance model Centropages typicus in the Bay of Biscay over the period 1970-2012.***

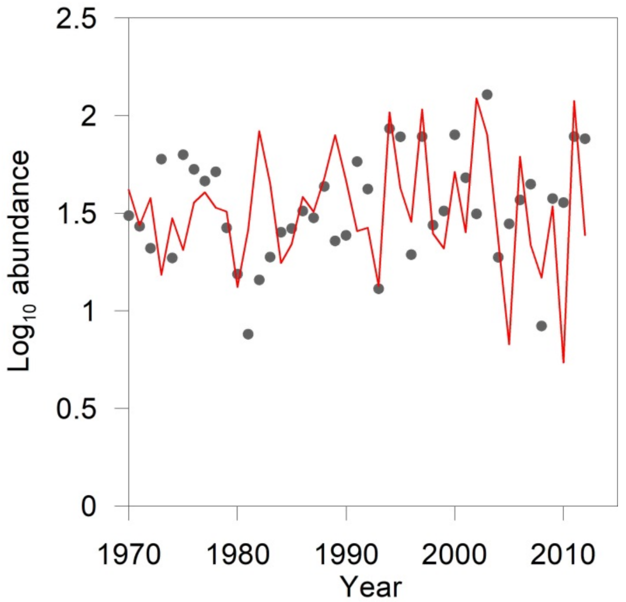

Year-to-year abundance changes of *Centropages typicus*. Comparison of the observed data, in dots, and model output (see following equation and table), red continuous line.

$N_{plk,\,t} = 0.19 \cdot \mathit{TEMP}_{BB\,t} - 0.01 \cdot NAO_{\,t} - 1.84$

Coefficients of the model *Centropages* abundance as function of NAO and SST.

	Param.	Std Error	t	p
Intercept	-1.84	0.15	-12.27	0.00
NAO	-0.01	0.02	-0.40	0.69
TEMP	0.19	0.01	19.25	0.00

Explanatory ability of the model

R	R^2	$F_{2,29}$	p
0.69	0.47	191.74	0.001

III. Selection of '*a*' in $n_{1,\,t+1} = a \cdot N_{fish,\,t} \cdot \mathit{overlap}_{\,t}$ (see table 1).

1. ***For the Arcto-boreal fish***

We first found the relationship among *a*, *overlap*, N_{t+1} and N_{t} if the population is stable ($N_{t+2} = N_{t+1}$).

Following equation in Table 1 where

$N_{t+2} = (n_{t+1} + N_{t+1}) \cdot 0.5$

If $N_{t+2} = N_{t+1}$ then $n_{t+1} = N_{t+1}$

since $n_{t+1} = a \cdot N_t \cdot \mathit{overlap}_{\,t}$

this means that $N_{t+1} = a \cdot N_{t} \cdot \mathit{overlap}_{\,t}$

and $a = N_{t+1} / (N_{t} \cdot \mathit{overlap}_{\,t})$

We then isolated two years when the abundance remained constant $N_{fish,t+2} = N_{fish,t+1}$ on the capelin data (i.e., 1980 and 1981) and extracted the values for the years t, t+1 and t+2 for N_{plk}, m_{plk}, and m_{fish} (given by the equations in table 1) and N_{fish} (Table 9.5 in ICES (ICES 2015)).

We then wrote a loop calculating the value of '*a*' for different value of *overlap* (ranging from 0.5 to 1 with a step of 0.01, assuming that the population cannot be maintained constant if the overlap is lower than 50%). We obtained 51 pairs of overlap and '*a*' values that were fitting the criteria. To select only one, we assume that a similar overlap at equilibrium as found for the temperate fish (0.62 see later). We obtained $a = 2.27$.

2. ***For the temperate fish***

Following equation in Table 1 where

$N_{t+1} = n_{t+1} + (N_t) \cdot 0.5$

If $N_{t+1} = N_t$ then $n_{t+1} = N_t \cdot 0.5$

since $n_{t+1} = a \cdot N_t \cdot overlap_t$

this means that $N_t \cdot 0.5 = a \cdot N_t \cdot overlap_t$

and $a = 1 / (2 \cdot overlap_t)$

We then wrote a loop calculating the standard deviation (sd) of the simulated overlap time series for a range of '*a*' values (0.5 to 1.5) and extracted the value of '*a*' corresponding to the sd of the anchovy data $\ln(n_{t+1}/N_t)$ (ICES 2011). The value corresponding to the anchovy recruitment sd was $a = 0.8$ giving an equilibrium of abundance when $overlap = 1/(2 \cdot 0.8) = 0.62$.

Supplementary tables

Table S1. Historical environmental variables.

Factor	Indices considered	Description and source
Temperature	$TEMP_t$	Barents Sea (BS) monthly mean temperatures at 0-200 m depth in Atlantic water parts of the Kola section (70.5 - 72.5°N, 33.5°E) over 1921-2010 [a]
		Bay of Biscay (BB) monthly mean sea surface temperatures extracted over the distribution area (Long 43.8°N to 47.8°N/ Lat 4.22°W to 7.87°W) [b]
Seasonal temperature	$TEMP_{SPR, t}$ $TEMP_{SUM, t}$	Mean BS temperature for March $_t$ to May $_t$, and June $_t$ to Aug $_t$ [a]
Annual temperature	$TEMP_{KOLA, t}$	Mean BS temperature for and Jan $_t$ to Dec $_t$
Annual temperature	$TEMP_{BB, t}$	Mean BB temperature for and Jan $_t$ to Dec $_t$
Temperature change	$TEMP_{\Delta SUM, t}$	temperature change from spring to summer ($TEMP_{\Delta SUM, t} = TEMP_{SUM, t} - TEMP_{SPR, t}$) for BS[a]
North Atlantic Oscillation	NAO_t	Station based winter (Dec $_{t-1}$ – March $_t$) NAO index[c]

Subscript *t* refers to year.

[a] http://www.pinro.ru/

[b] from the extended reconstructed SST (NOAA_ERSST_V3) data set provided by the Physical Sciences Division of the NOAA Earth System Research Laboratory. Data are available from 1960 to 2013 with 1 × 1 degree grid resolution. www.esrl.noaa.gov/psd/

[c] https://climatedataguide.ucar.edu/climate-data/hurrell-north-atlantic-oscillation-nao-index-station-based

Table S2. Summary of the sensitivity analyses.

Variable tested	Arcto-boreal	Temperate fish
Temperature	+24 %	-15 %
NAO	+20 %	- 20 %
N_{plk}	+42 %	+3.3 %

Change in N_{fish} in % due to the increase of a selected variable affecting the model by half a standard deviation from 1960 (to have the same duration of change for TB and AB).
Note that plankton abundance N_{plk} is also affected by the change of temperature (Table 2) and that the effect of temperature on N_{fish} is thus the sum of the direct temperature effect on the timing of predators and prey and the indirect effect through N_{plk}.
Sensitivity analyses show that, for the Arcto-Boreal biome, the fish abundance is positively affected by an increase of half a standard deviation, during the historical period, of temperature (corresponding to ca 0.25 °C), of NAO (ca +1), and N_{plk} (ca 0.33).
For the temperate biome, the fish abundance is positively affected by an increase of N_{plk} (ca 0.04) but negatively affected by an increase of temperature (ca 0.25 °C) and NAO (ca +1).

Supplementary Figures

Figure S1. Historical and projected temperature change by a high emissions scenario.

Historical temperature change (before 2013) and projected temperature change by a medium emissions scenario (RCP4.5, radiative forcing of 4.5 W m^{-2} at year 2100 relative to pre-industrial conditions) for the Barents Sea (Kola section, plain line) and the Bay of Biscay (red dashed line). The projection model was run three times giving slightly different day to day change as presented in the three different plots. Note different temperature scales for Barents Sea and Bay of Biscay. Note that the yearly standard deviation for the historical period is higher in the Temperate (Bay of Biscay) than in the Arcto-boreal (Barents Sea) seas (2.94 °C ± 0.24 vs. 0.75 °C ± 0.14 respectively for Run 1).

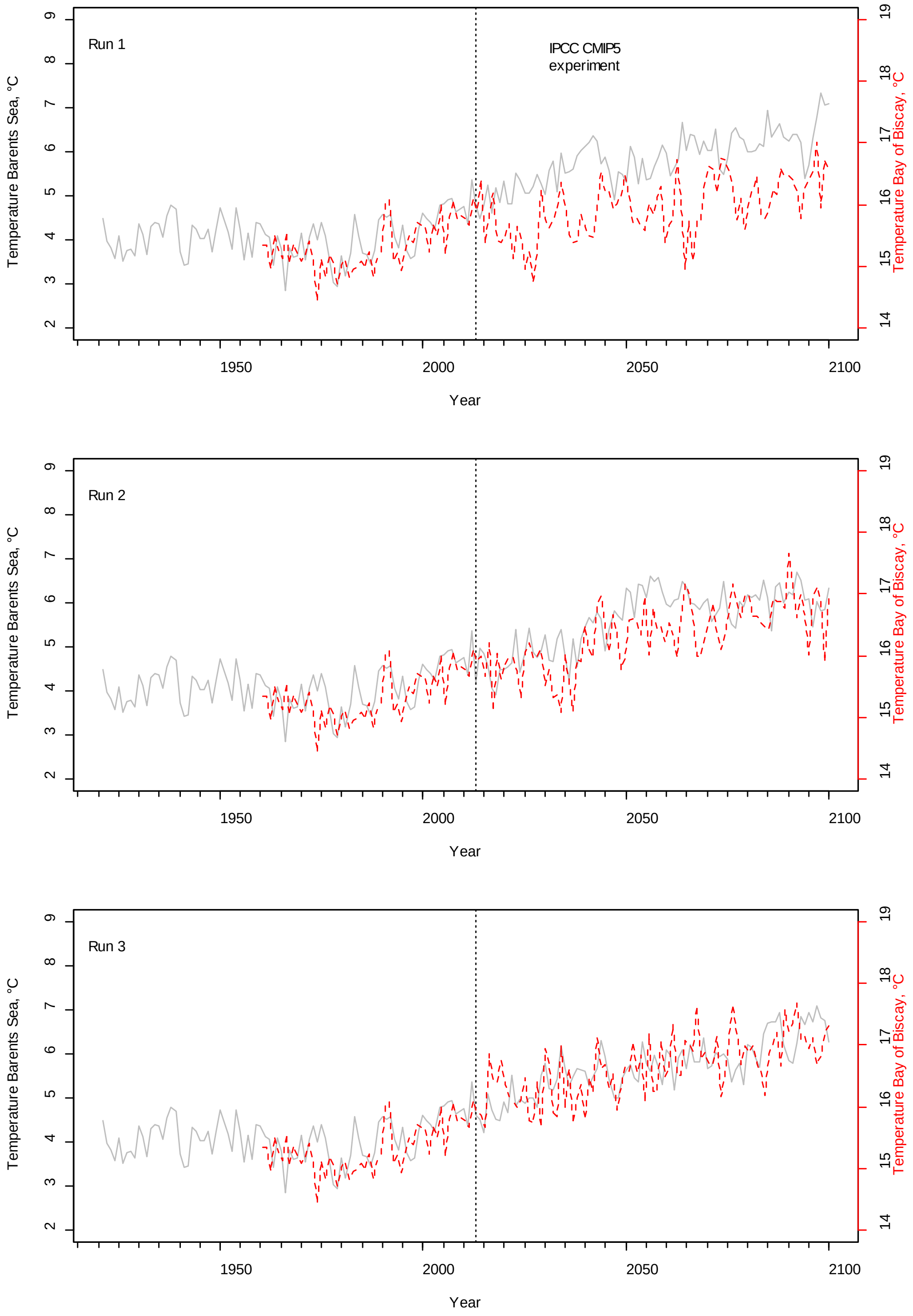

Figure S2. Change of the overlap between the historical period (before 2013) and the projection period (after 2013) for both biomes.

The frequency of Overlap was calculated on the average of each 3 runs for the projection period. The figure shows that the Arcto-boreal fish will be confronted during the projection period to more frequent full mismatch (and match) than during the historical period while the Temperate fish globally experienced the same frequency of overlaps.

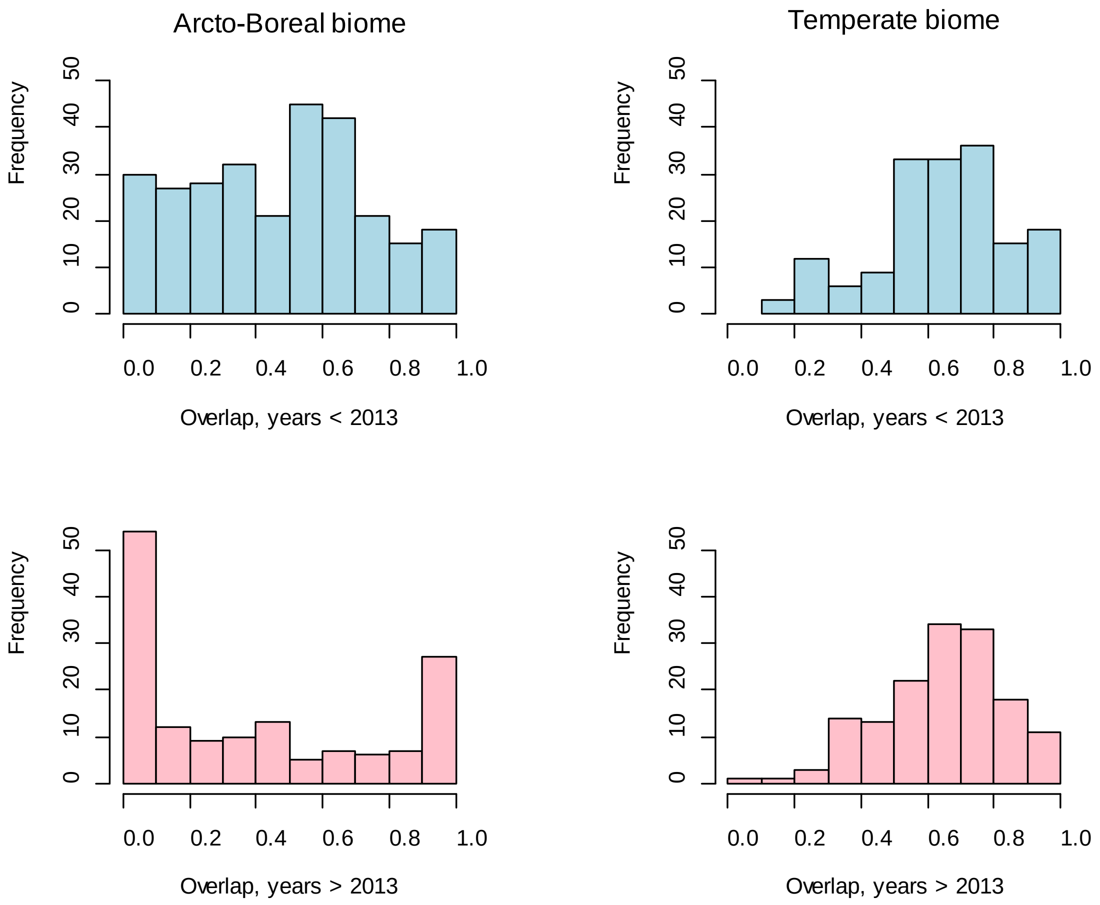

Figure S3. Sea temperature and spawning of the Barents Sea capelin (*Mallotus villosus*) 1951-1961. Spawning time (blue crosses) comes from Table 1 (Gjøsæter 1972) and temperature in °C from the Kola section (see Supplementary Table S2). Over-imposed are the curves resulting from a 2nd order polynomial equation fitted each year to the monthly temperature values. The red cross is the lowest temperature of each curve to compare to the date of spawning (respectively: mean of 3.12 ± 0.42 in months vs. 3.27 ± 0.39).

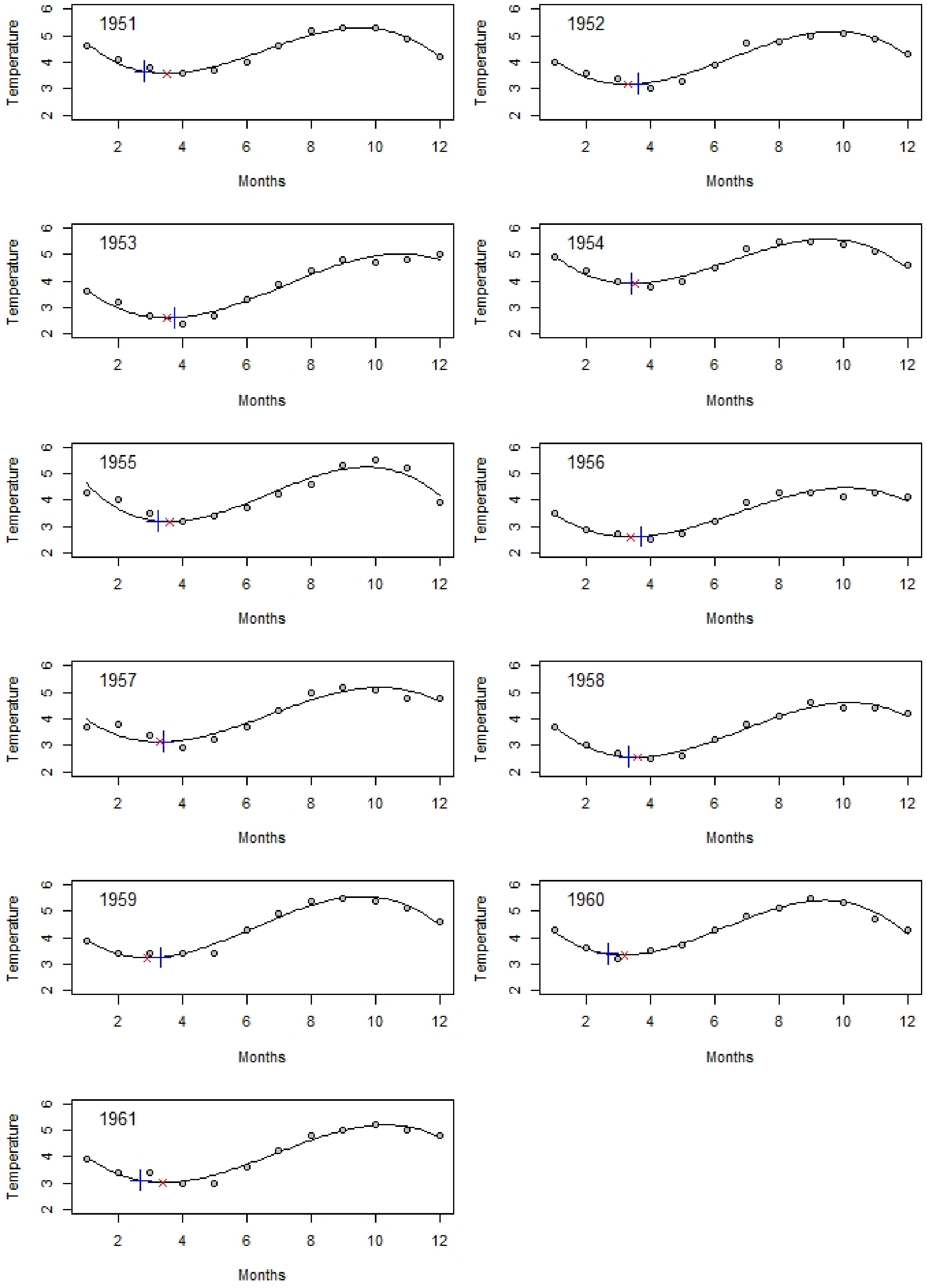

Figure S4. Effect of stochasticity on the survival terms for the simulation and the projection.

Simulation and projection (RCP4.5, radiative forcing of 4.5 W m^{-2} at year 2100 relative to pre-industrial conditions, run 1). Dashed lines are the reported results, with dots for the simulation before 2013. The shaded areas come from a bootstrap procedure of 500 replicates. 0.05 and 0.95 quantiles are displayed. Blue shaded areas are with stochasticity applied on the overlap level only. Pink shaded area are for stochasiticity apply to the overlap level and survival (table 1). Stochasticity was modelled using *rnorm* function with a sd=0.1.

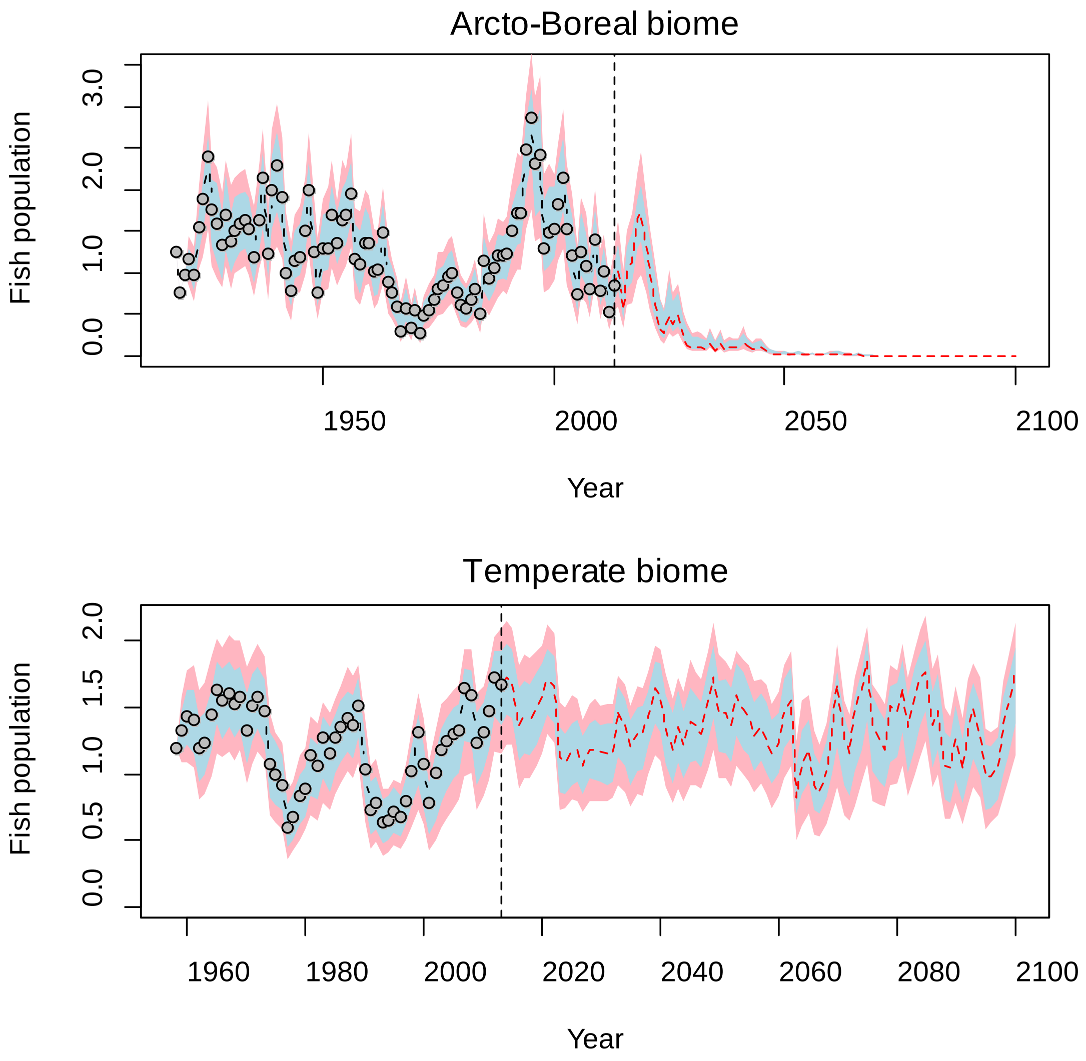

Figure S5. Comparison of the model using a normal against skewed (log normal) distribution. Comparison of the model using a normal distribution (dots) and log normal distribution (dashed lines) for the Arcto-boreal fish (left) and for the temperate fish (right) population. Each plot presents the simulation for one single run for the medium emissions scenario (RCP4.5). Two level of skewness for the log-normal distribution are used (standard deviation of the variable's natural logarithm = 0.20 — dashed blue lines, and sd = 0.25 — dotted red lines). Over sd = 0.25 level of skewness the distribution will be ranging over more than 3 months that makes limited biological meaning. A skewed distribution buffers the effect the increase of the temperature has on the fish abundance dynamics for both systems but the general pattern remains the same: decrease to collapse for the Arcto-boreal fish and maintenance for the temporal fish abundance.

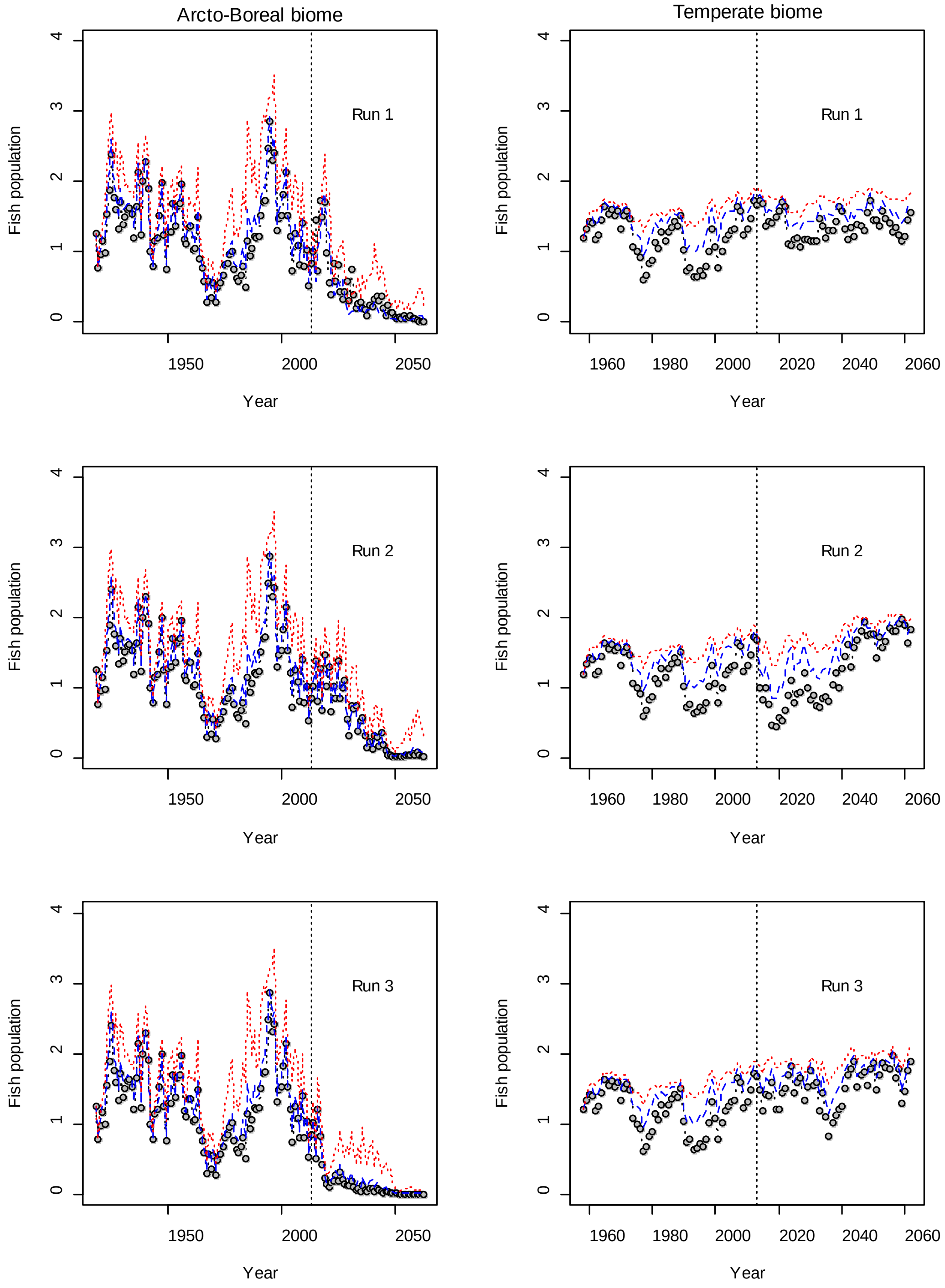

Figure S6. Simulation with full synchrony. In dots is presented the simulation as given in the text. The dashed blue line is the simulation for a total temporal match (m_{fish}=m_{plk}) change for the Arcto-boreal fish in the in the Barents Sea and the temperate fish in the Bay of Biscay.

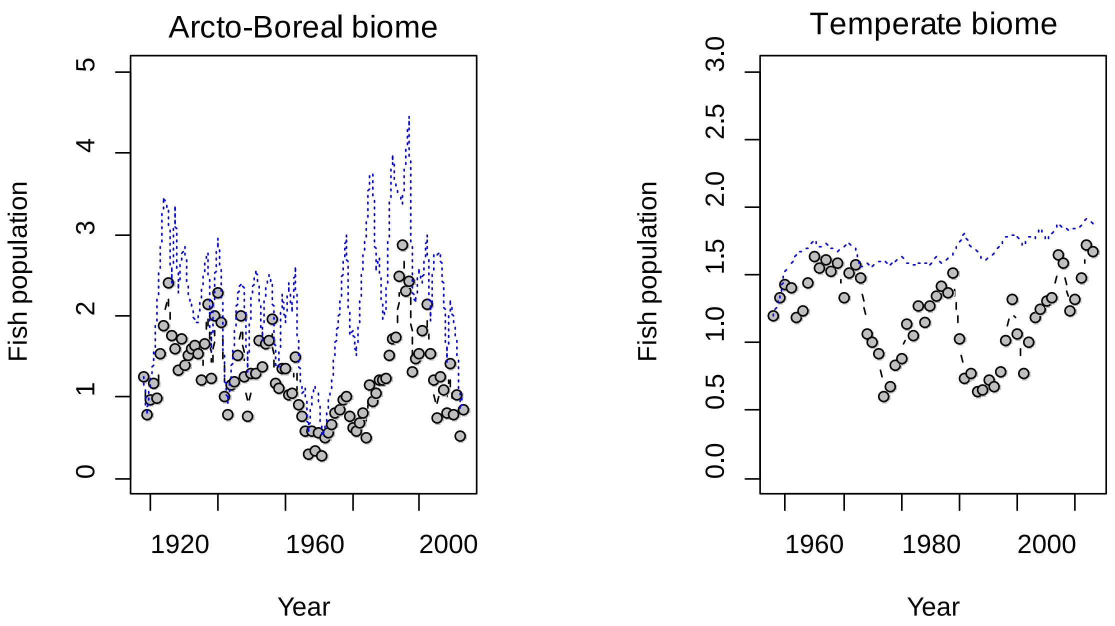

Figure S7. Effect of the relative importance of the match-mismatch on young production for the projection period.

We used the run 1 of the RCP4.5 (radiative forcing of 4.5 W m^{-2} at year 2100 relative to pre-industrial conditions) for the projection. The importance of the Overlap (Table 1) was reduced from 100 % (our model, dotted line, see fig. 2) to 55 %, the remaining part being modelled as a full match (note that it means that there was no density-dependence or predation for the non-match-mismatch part). The figure shows that the projected decrease of the Arcto-boreal fish still occurs with a match-mismatch relationship of at least 60%. In a similar way, without a match-mismatch relationship of at least 70% the Temperate fish population increases.

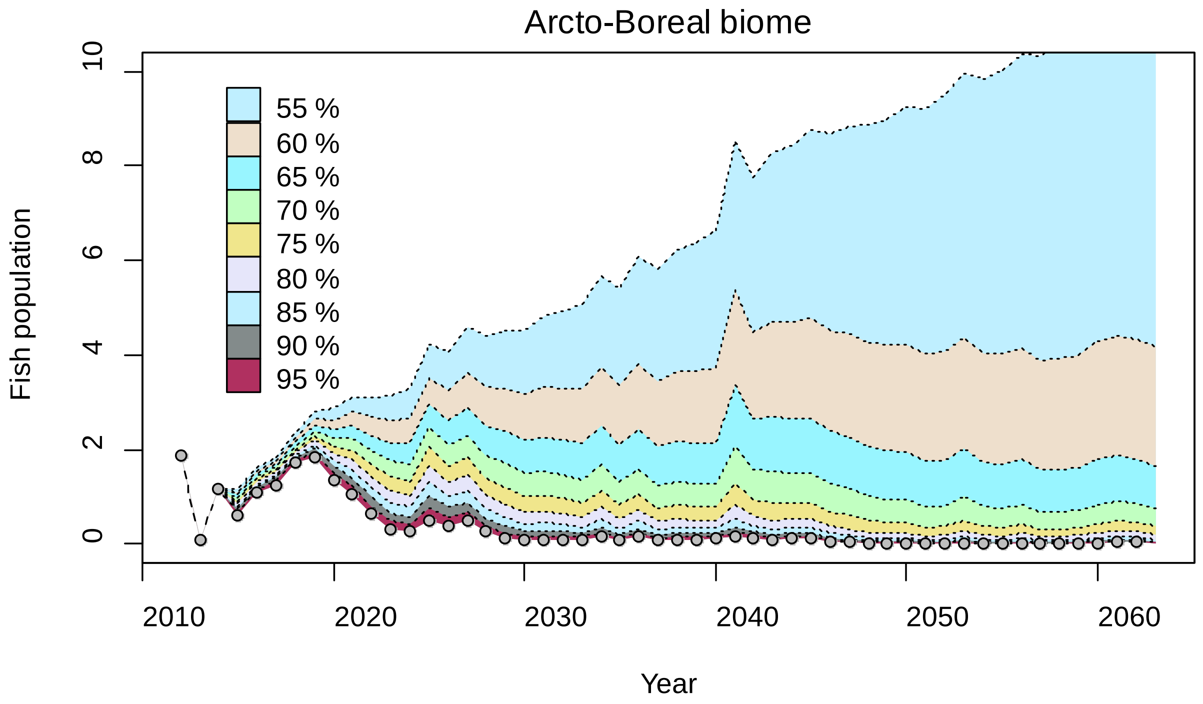

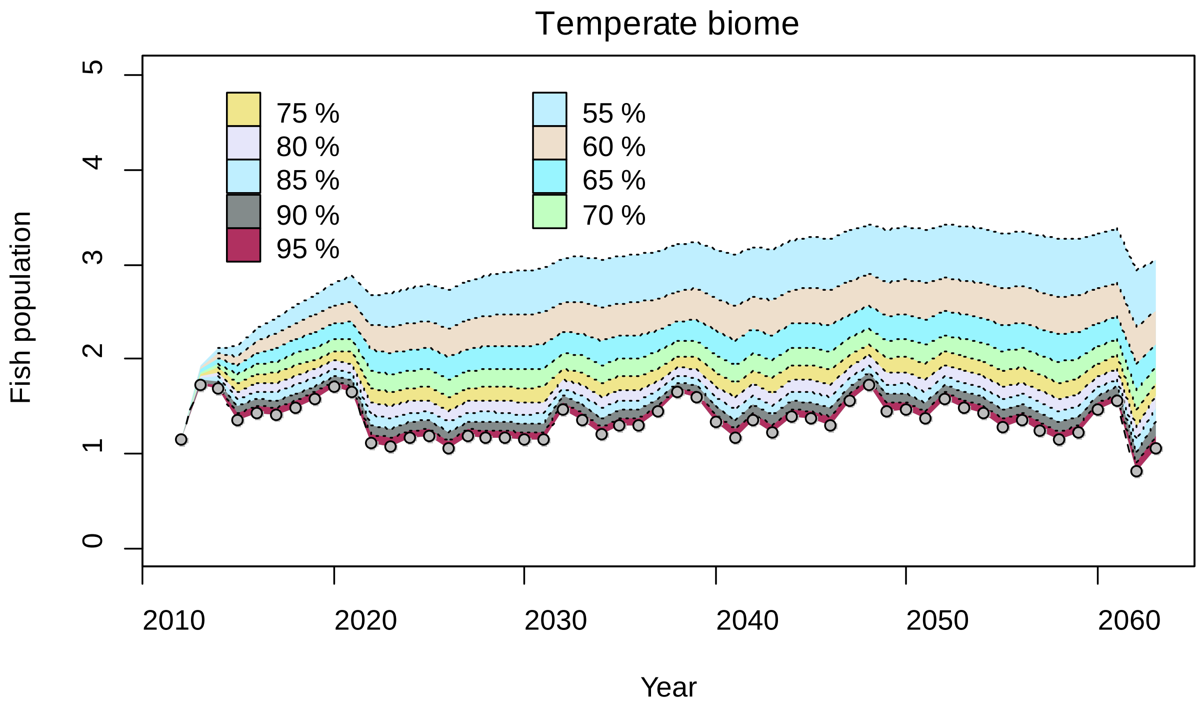

Figure S8. Plasticity adaption of the Arcto-boreal fish to warmer climate. Effect of a life cycle for the Arcto-boreal fish in the in the Barents Sea reduced to one year (dashed blue line) compared to the two-year life cycle of the model (dots).

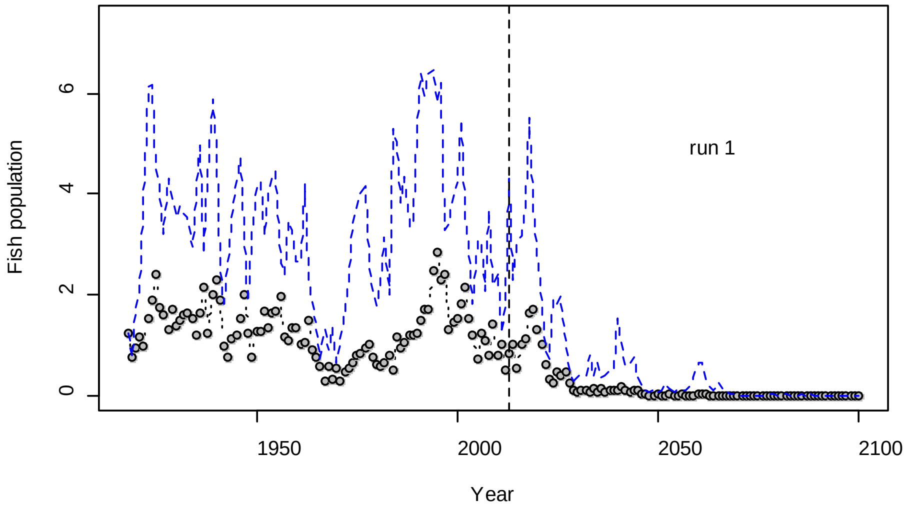

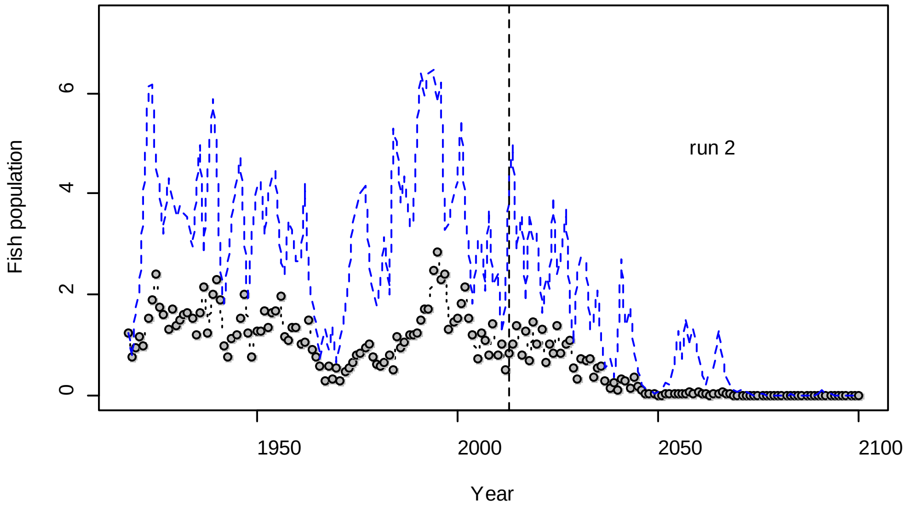

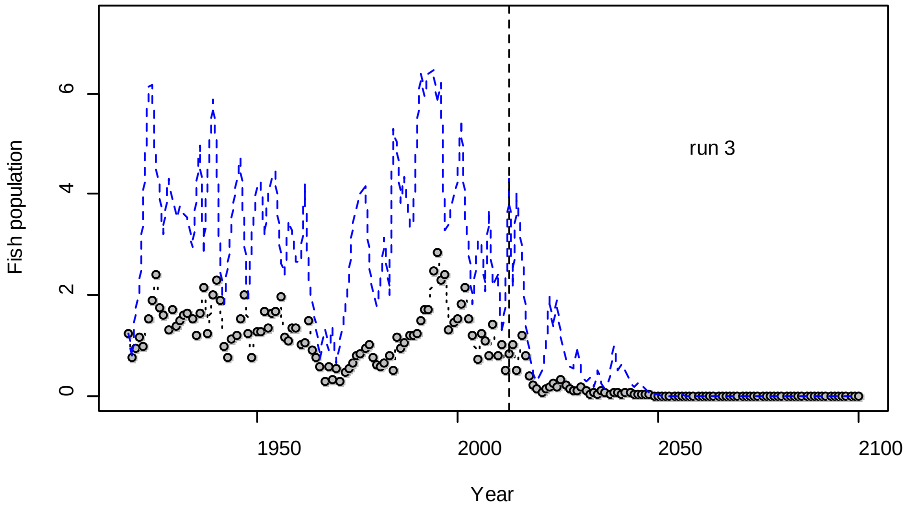

Figure S9. Relationship between environmental variables and the variables entering the model. Relationship between temperature and the three variables entering the normal equation used to calculate the overlap (Table 2: m_{plk}, N_{plk} and m_{fish}) for the Arcto-boreal fish in the in the Barents Sea (left column) and the temperate fish in the Bay of Biscay (right column). The associations are based on temperatures and modelled dynamics in the pre-2013 period.

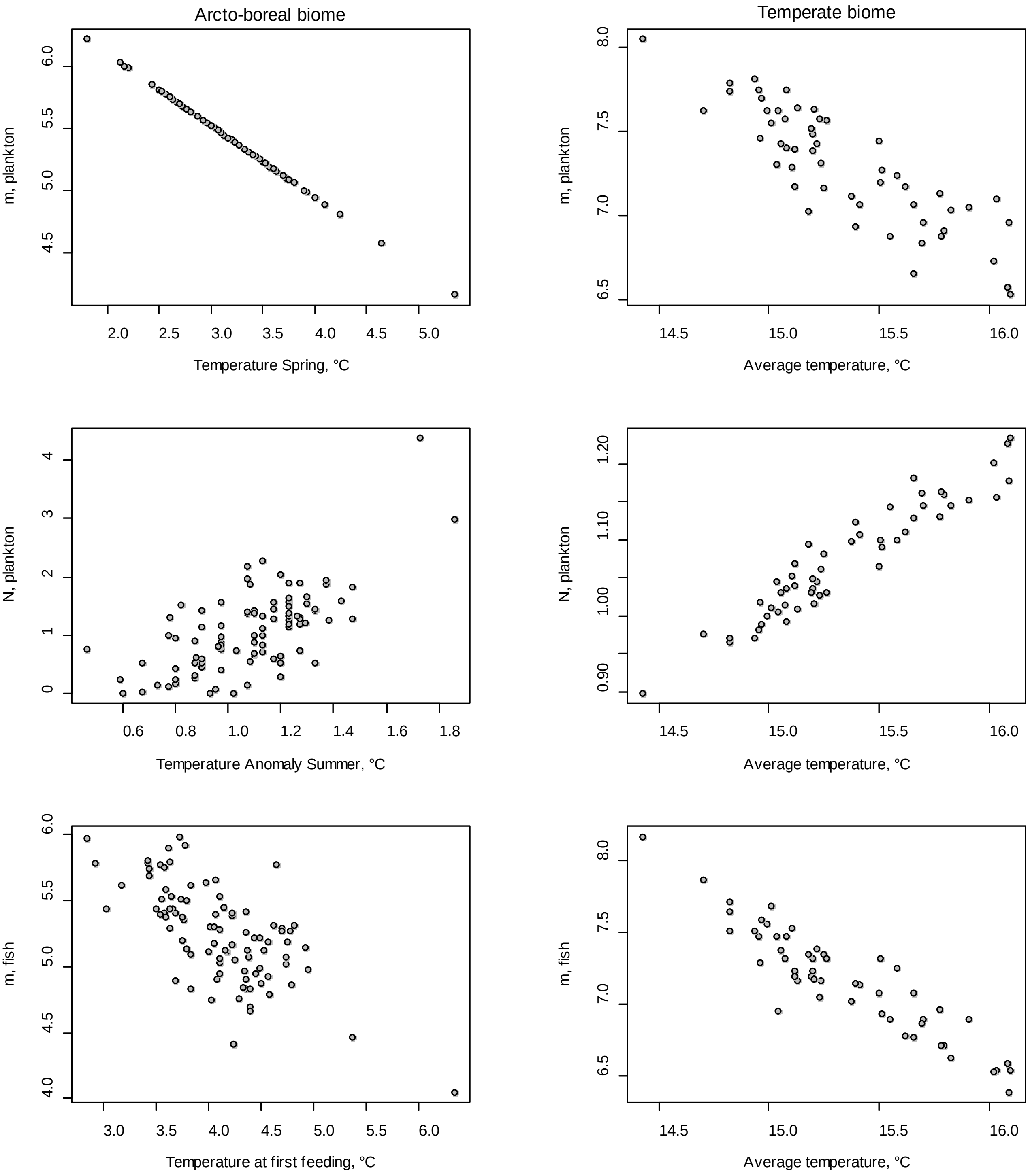

References

Cushing, D. H. 1990. Plankton production and year-class strength in fish populations - an update of the match mismatch hypothesis. Advances in Marine Biology 26:249-293.

Durant, J. M., D. O. Hjermann, T. Anker-Nilssen, G. Beaugrand, A. Mysterud, N. Pettorelli, and N. C. Stenseth. 2005. Timing and abundance as key mechanisms affecting trophic interactions in variable environments. Ecology Letters 8:952-958.

Durant, J. M., D. O. Hjermann, T. Falkenhaug, D. J. Gifford, L. J. Naustvoll, B. K. Sullivan, G. Beaugrand et al. 2013. Extension of the match-mismatch hypothesis to predator-controlled systems. Marine Ecology Progress Series 474:43-52.

Durant, J. M., D. O. Hjermann, G. Ottersen, and N. C. Stenseth. 2007. Climate and the match or mismatch between predator requirements and resource availability. Climate Research 33:271-283.

Gjøsæter, J. 1972. Recruitment of the Barents Sea capelin 1951-1961. ICES Council meeting H24:1-9.

ICES. 2011. Report of the Working Group on Anchovy and Sardine (WGANSA), 24-28 June 2011, Vigo, Spain. ICES CM 2011/ACOM 16:pp. 470.

—. 2015. Report of the Arctic Fisheries Working Group (AFWG) 23 - 29 April 2015, Hamburg, Germany. ICES CM 2015/ACOM 05:639 pp.

Reid, P. C., J. M. Colebrook, J. B. L. Matthews, J. Aiken, and T. Continuous Plankton Recorder. 2003. The Continuous Plankton Recorder: concepts and history, from plankton indicator to undulating recorders. Progress in Oceanography 58:117-173.
